# Supplementary material for: Huntingtin fibrils with different toxicity, structure, and seeding potential can be interconverted
Source: Nat Commun. 2021 Jul 13;12:4272. doi: 10.1038/s41467-021-24411-2 (PMC8277859; doi:10.1038/s41467-021-24411-2)
Supplement: Supplementary file 1 — Supplementary Information [file 41467_2021_24411_MOESM1_ESM.pdf]

# Supplementary Information for

# Huntingtin fibrils with different toxicity, structure, and seeding potential can be interconverted

**J. Mario Isas[1], Nitin K. Pandey[1], Hui Xu[1], Kazuki Teranishi[1], Alan K. Okada[1,2], Ellisa K. Fultz[1], Anoop Rawat[1], Anise Applebaum[1], Franziska Meier[1], Jeannie Chen[1], Ralf Langen[1*], and Ansgar B. Siemer[1*]**

1. Department of Physiology & Neuroscience, Zilkha Neurogenetic Institute, Keck School of Medicine, University of Southern California
2. Current Address: Regions Hospital, Department of Emergency Medicine, St. Paul, MN

Email: langen@usc.edu, asiemer@usc.edu

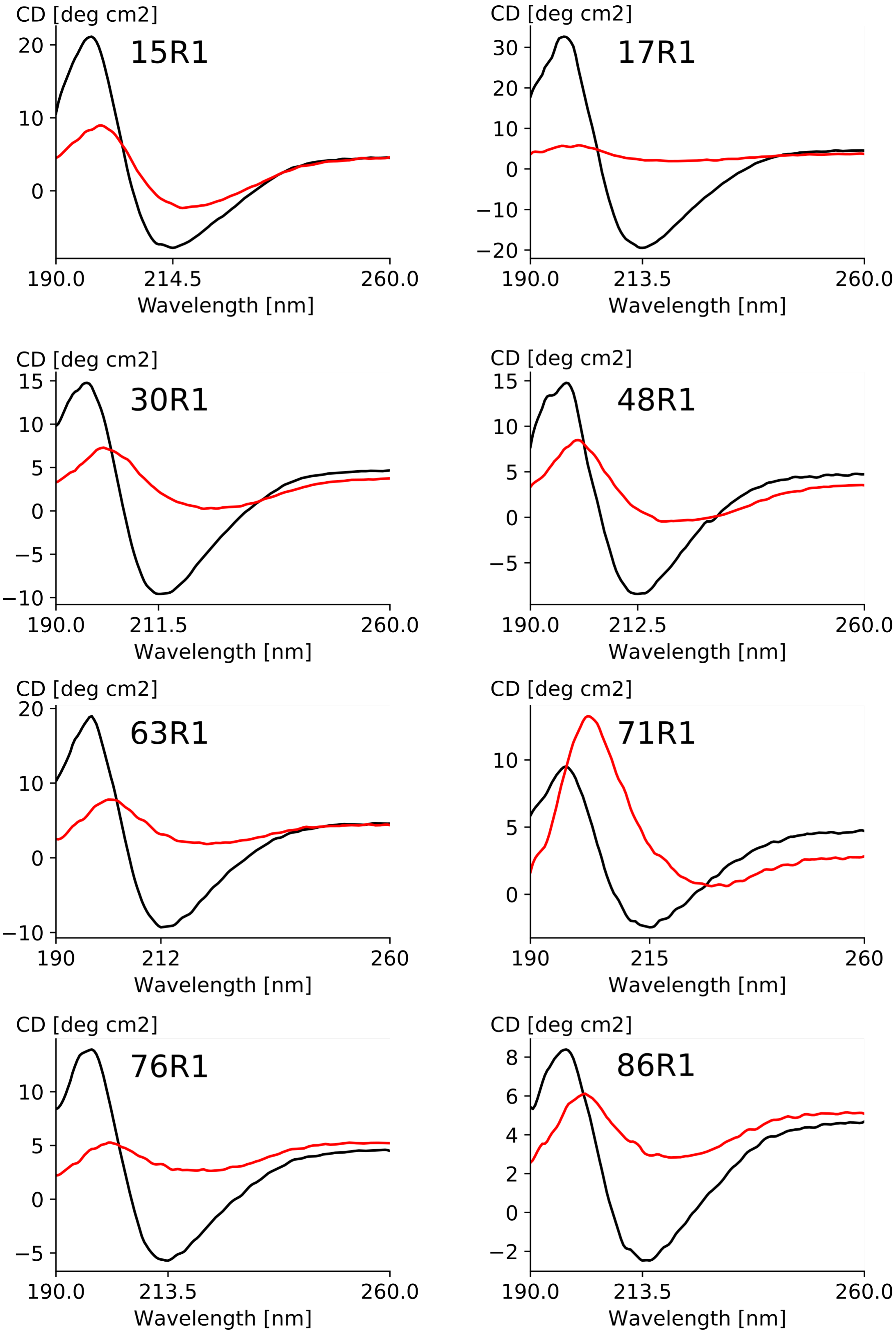


**Supplementary Figure 1:** CD spectra of all the MTSL labeled HTTex1 samples used for the EPR data of Figure 2. The T-fibril spectra (black) show typical minima around 213 nm whereas all the N-fibril spectra (red) are red shifted and are of decreased intensity because the bundling of the fibrils increases scattering.

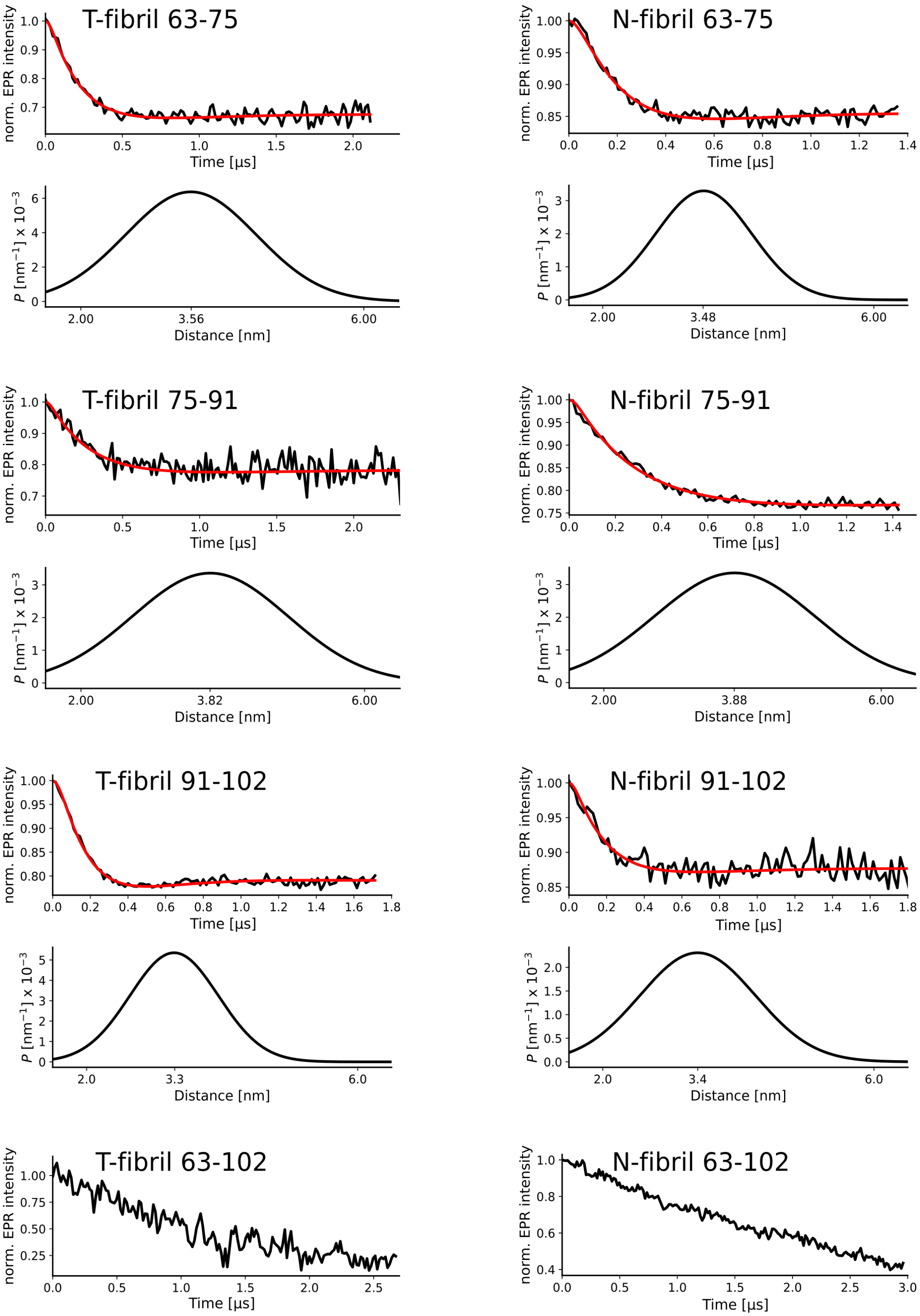


**Supplementary Figure 2:** DEER data and fits corresponding to the distances reported in Figure 5. Top panels: base line corrected DEER data (black) and fit to single Gaussian distribution (red). Bottom panels: Gaussian distribution corresponding to fit. The center of the distribution is indicated. Because the distance between residues 63-102 was above the detection limit, no fit is shown and no baseline was subtracted in this case.

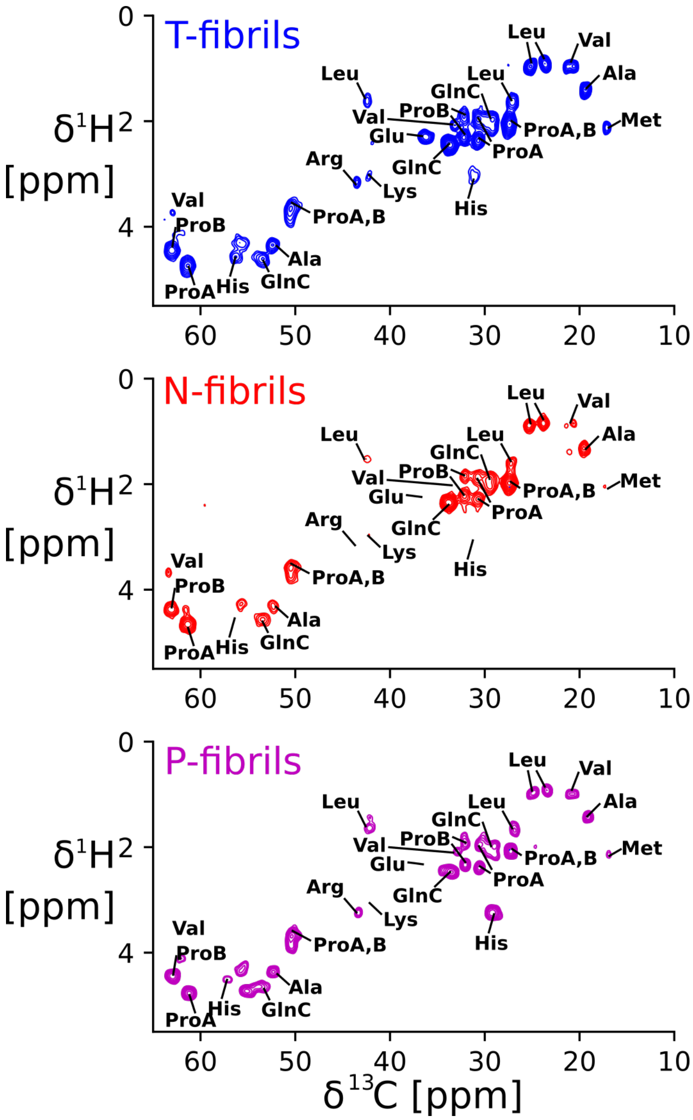


**Supplementary Figure 3:** INEPT HETCOR 2D $^{1}H$-$^{13}C$ spectra of the three $HTT_{ex1}Q46$ fibril types show very little chemical shift changes. Notable differences include the absence of His shifts in the N-fibrils and the shift of the corresponding peaks in the protofibril spectra due to a change in pH. While the extent of dynamics in the C-termini varies between the different fibril types, their chemical shift does not change.

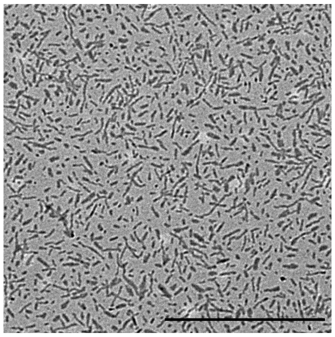

**Supplementary Figure 4:** EM of P-fibrils made from T-fibrils via treatment with 0.5% TFA and sonication. This treatment is highly reproducible and has let to similar results dozen of times. Scale bars denotes 1 µm.

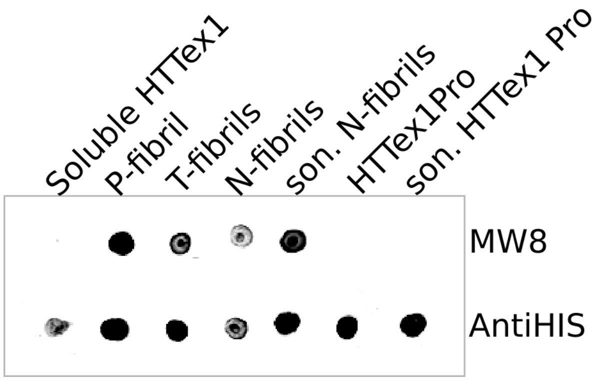


**Supplementary Figure 5:** The exposed PRD of the T and P-fibrils is better accessible PRD specific antibodies than the PRD of the N-fibrils. Dot blot of HTTex1 monomer and different HTTex1 fibril types. The MW8 and fibril specific antibody that recognizes an epitope in the PRD and an His tag antibody were used for detection. MW8 strongly binds to P and T-fibrils and to a lesser degree N-fibrils. Sonication of N-fibrils (son. N-fibril) significantly increases the binding of MW8. The HTTex1 monomer and fibrils formed by a HTTex1 mutant lacking the MW8 epitope of the PRD (HTTex1Pro) show no binding to MW8. These data were reproduced twice.

**Supplementary Table 1:** Primers used in this study.

| | |
|---|---|
| 86R1 forward | cca cag gca cag cct tgc ctg cct cag cca caa |
| 86R1 reverse | ttg tgg ctg agg cag gca agg ctg tgc ctg tgg |
