# Supplementary material for: Huntingtin fibrils with different toxicity, structure, and seeding potential can be interconverted
Source: Nat Commun. 2021 Jul 13;12:4272. doi: 10.1038/s41467-021-24411-2 (PMC8277859; doi:10.1038/s41467-021-24411-2)
Supplement: Supplementary file 3 — Reporting Summary [file 41467_2021_24411_MOESM3_ESM.pdf]

nature research

Corresponding author(s): Ansgar B. Siemer

Last updated by author(s): 06/10/2021

Reporting Summary

Nature Research wishes to improve the reproducibility of the work that we publish. This form provides structure for consistency and transparency in reporting. For further information on Nature Research policies, see our Editorial Policies and the Editorial Policy Checklist.

Statistics

For all statistical analyses, confirm that the following items are present in the figure legend, table legend, main text, or Methods section.

n/a	Confirmed	
☐	☒	The exact sample size (*n*) for each experimental group/condition, given as a discrete number and unit of measurement
☐	☒	A statement on whether measurements were taken from distinct samples or whether the same sample was measured repeatedly
☐	☒	The statistical test(s) used AND whether they are one- or two-sided<br>*Only common tests should be described solely by name; describe more complex techniques in the Methods section.*
☒	☐	A description of all covariates tested
☒	☐	A description of any assumptions or corrections, such as tests of normality and adjustment for multiple comparisons
☐	☒	A full description of the statistical parameters including central tendency (e.g. means) or other basic estimates (e.g. regression coefficient) AND variation (e.g. standard deviation) or associated estimates of uncertainty (e.g. confidence intervals)
☐	☒	For null hypothesis testing, the test statistic (e.g. *F*, *t*, *r*) with confidence intervals, effect sizes, degrees of freedom and *P* value noted<br>*Give P values as exact values whenever suitable.*
☒	☐	For Bayesian analysis, information on the choice of priors and Markov chain Monte Carlo settings
☒	☐	For hierarchical and complex designs, identification of the appropriate level for tests and full reporting of outcomes
☒	☐	Estimates of effect sizes (e.g. Cohen's *d*, Pearson's *r*), indicating how they were calculated

Our web collection on statistics for biologists contains articles on many of the points above.

Software and code

Policy information about availability of computer code

Data collection	NMR: Agilent VNMRJ 4.0; EPR: Bruker WinEPR 4.33; EM: Gatan DigitalMicrograph 1.85.1535; CD: Jasco Spectrum Measurement;
Data analysis	NMR: NMRpipe 9.4, nmrglue 0.7; EPR: EPR130, DEER Analysis 2019; EM: ImageJ 1.52; Microscopy: ImageJ 1.52; CD: Python 3.7.3, SciPy 1.1.0, NumPy 1.16.2

For manuscripts utilizing custom algorithms or software that are central to the research but not yet described in published literature, software must be made available to editors and reviewers. We strongly encourage code deposition in a community repository (e.g. GitHub). See the Nature Research guidelines for submitting code & software for further information.

Data

Policy information about availability of data

All manuscripts must include a data availability statement. This statement should provide the following information, where applicable:

- Accession codes, unique identifiers, or web links for publicly available datasets
- A list of figures that have associated raw data
- A description of any restrictions on data availability

Source data are provided with this paper. All other data are available from the corresponding authors upon reasonable request.

Field-specific reporting

Life sciences study design

All studies must disclose on these points even when the disclosure is negative.

Sample size	For our cell work, no statistical method was used to determine sample size. At least three biological replicates were measured for each condition so that a standard deviation could be calculated. P-values indicate that the number of replicates was sufficient. EPR aggregation kinetics were measured with three biological replicates to ensure reproducibility.
Data exclusions	No data were excluded
Replication	The cell data were repeated at least three times. EPR, NMR, and CD spectroscopic experiments were only measured once. However, we like to point out that the EPR experiments were repeated with spin labels at different conditions and the CD controls were measured for each of these repeats. All of these repetitions were successful. EM images were repeated multiple times, all repeats were successful. The dot blot was repeated twice, both repeats were successful.
Randomization	Samples were not randomized. Randomization was not possible because the fibril polymorphs can be told apart just by looking at them (see Figure 1). In addition, no covariates could be identified in our cell based work.
Blinding	The analysis of cell seeding and cell toxicity data was blinded. The data collection was not blinded because the fibril polymorphs can be told apart just by looking at them (see Figure 1). The data collection and analysis of the other data was not blinded. Blinding was not necessary because the effects in our biophysical measurements were often large or blinding was not feasible because progression of the same sample though different conditions or time was monitored.

Reporting for specific materials, systems and methods

We require information from authors about some types of materials, experimental systems and methods used in many studies. Here, indicate whether each material, system or method listed is relevant to your study. If you are not sure if a list item applies to your research, read the appropriate section before selecting a response.

Materials & experimental systems

n/a	Involved in the study	
☐	☒	Antibodies
☐	☒	Eukaryotic cell lines
☒	☐	Palaeontology and archaeology
☒	☐	Animals and other organisms
☒	☐	Human research participants
☒	☐	Clinical data
☒	☐	Dual use research of concern

Methods

n/a	Involved in the study	
☒	☐	ChIP-seq
☒	☐	Flow cytometry
☒	☐	MRI-based neuroimaging

Antibodies

Antibodies used	MW8 was a gift from Ali Khoshnan. It can be purchased from Sigma-Aldrich Cat. No. MABN2529. Anti-polyHisitinde was purchased from Sigma-Aldrich Ca. No. OB05. Li-Cor Goat anti-Mouse IgG P/N 92532210 Lot# C61012-06.
Validation	MW8 was validated via its epitope (HTTex1 fibrils) in Figure S4. Anti-polyHistidine was tested with a positive control i.e. proteins containing a His-Tag sequence by its manufacturer.

Eukaryotic cell lines

Policy information about cell lines

Cell line source(s)	Neuro-2a cells were purchased from ATCC, ST HDH Q7/111 cell line was purchased from the Coriell Institute (Cat #: CH00096)
Authentication	Neuro-2a cells were authenticated by ATCC using karyotyping. ST HDH Q7/111 cells were authenticated by the Coriell Institute by PCR genotyping of the Q length.
Mycoplasma contamination	Cell lines was not tested for Mycoplasma contamination in house but by previously by ATCC and Coriell Institute
Commonly misidentified lines (See ICLAC register)	No commonly misidentified lines were used in this study.
